# A Retrospective Study of Retired Academics: Long-term Career Benefits of Past Mentoring Behaviors

**DOI:** 10.1093/geroni/igab046.2826

**Published:** 2021-12-17

**Authors:** Giovanna Garrido Blanco, Jordan Boeder, Veronica Fruiht, Kevin Erikson, Sarah Hwang, Thomas Chan

**Affiliations:** 1 California State University Northridge, Chanhassen, Minnesota, United States; 2 California State University Northridge, Northridge, California, United States; 3 Dominican University of California, Dominican University of California, California, United States

## Abstract

While there is extensive literature on the benefits of mentoring for mentees, less is known about the impact of those relationships on mentors, particularly, after mentors complete their careers (i.e., retirement). For academics, the time and energy spent mentoring students can either be beneficial or costly to productivity. This study explores the associations between past mentoring and present evaluations of retired academics’ careers, seeking to investigate the long-term career benefits of mentoring. Understanding the evaluation of older adults’ careers at retirement in relation to their prior engagement in mentoring is critical, as mentoring is an integral component of careers in academia. Survey data were collected from a national sample of 277 retired academics averaging 73.6 (SD=6.2) years old and 7.7 (SD=5.8) years post-retirement. Results from structural equation models demonstrated that retired academics who reported having more protegees (β=.19, p=.024) and engaged in more mentoring behaviors (β=.18, p=.027) exhibited increased objective career benefits. Providing more mentoring functions was also associated with higher subjective career achievement (β=.33, p<.001). Interestingly, the number of mentees and mentoring behaviors were not correlated to career satisfaction. Findings from the current study demonstrate the association between past mentoring experiences with career success. Examining the link between mentoring behaviors and overall assessments of career in retirement offers important insight into the long-term benefits of mentoring in higher education training prompting further research into the realization of these benefits in later life.

